# Nitrous Oxide—Its Properties—Methods of Administration and Effects

**Published:** 1888-02

**Authors:** 


NITROUS OXIDE. ITS PROPERTIES. METHODS OF ADMINISTRATION AND EFFECTS. By S. H. Guilford, A. M., D. D. S

Prof. Guilford has furnished a manual which will prove of great value to the student, and also to the practitioner, as it is based on practice, experience and observations in the use of nitrous oxide. Full directions for the administration of this gas, and the extraction of teeth under its influence and measures for resuscitation are given with great exactness, and render this work a necessity in the dental office. Philadelphia, 1887.

